# Precision Medicine: What Challenges Are We Facing?

**DOI:** 10.1016/j.gpb.2016.10.001

**Published:** 2016-10-13

**Authors:** Yu Xue, Eric-Wubbo Lameijer, Kai Ye, Kunlin Zhang, Suhua Chang, Xiaoyue Wang, Jianmin Wu, Ge Gao, Fangqing Zhao, Jian Li, Chunsheng Han, Shuhua Xu, Jingfa Xiao, Xuerui Yang, Xiaomin Ying, Xuegong Zhang, Wei-Hua Chen, Yun Liu, Zhang Zhang, Kun Huang, Jun Yu

**Affiliations:** 1 MOE Key Laboratory of Molecular Biophysics, College of Life Science and Technology and the Collaborative Innovation Center for Brain Science, Huazhong University of Science and Technology, Wuhan 430074, China; 2 School of Electronic and Information Engineering, Xi’an Jiaotong University, Xi’an 710049, China; 3 CAS Key Laboratory of Mental Health, Institute of Psychology, Chinese Academy of Sciences, Beijing 100101, China; 4 State Key Laboratory of Medical Molecular Biology, Institute of Basic Medical Sciences, Chinese Academy of Medical Sciences, Peking Union Medical College, Beijing 100005, China; 5 MOE/Beijing Key Laboratory of Carcinogenesis and Translational Research, Center for Cancer Bioinformatics, Peking University Cancer Hospital & Institute, Beijing 100142, China; 6 Center for Bioinformatics, State Key Laboratory of Protein and Plant Gene Research, School of Life Sciences, Peking University, Beijing 100871, China; 7 Beijing Institutes of Life Science, Chinese Academy of Sciences, Beijing 100101, China; 8 MOE Key Laboratory of Developmental Genes and Human Disease, Institute of Life Sciences, Southeast University, Nanjing 210096, China; 9 State Key Laboratory of Stem Cell and Reproductive Biology, Institute of Zoology, Chinese Academy of Sciences, Beijing 100101, China; 10 Max Planck Independent Research Group on Population Genomics, CAS-MPG Partner Institute for Computational Biology (PICB), Shanghai Institutes for Biological Sciences, Chinese Academy of Sciences, Shanghai 200031, China; 11 CAS Key Laboratory of Genome Sciences and Information, Chinese Academy of Sciences, Beijing 100101, China; 12 MOE Key Laboratory of Bioinformatics, School of Life Sciences, Center for Synthetic and Systems Biology, Tsinghua University, Beijing 100084, China; 13 Computational Omics Laboratory, Center of Computational Biology, Beijing Institute of Basic Medical Sciences, Beijing 100850, China; 14 Bioinformatics Division, TNLIST and MOE Key Laboratory for Bioinformatics, Department of Automation, Tsinghua University, Beijing 100084, China; 15 Department of Bioinformatics and Systems Biology, College of Life Science and Technology, Huazhong University of Science and Technology, Wuhan 430074, China; 16 MOE Key Laboratory of Metabolism and Molecular Medicine, Department of Biochemistry and Molecular Biology, School of Basic Medical Sciences, Fudan University, Shanghai 200032, China; 17 BIG Data Center and CAS Key Laboratory of Genome Sciences & Information, Beijing Institute of Genomics, Chinese Academy of Sciences, Beijing 100101, China; 18 Department of Biomedical Informatics, The Ohio State University, Columbus, OH 43017, USA

Following the publication of the US National Research Council (NRC) report “*Toward Precision Medicine: Building a Knowledge Network for Biomedical Research and a New Taxonomy of Diseases*” in 2011 [1], several nations have announced that their national research programs would definitely head toward this direction. Now, precision medicine (PM) became a banner for many large-scale biomedical research programs not only in the United States (US) but also in other nations including China. It is irrelevant now as to how much each nation wishes to or will invest on PM but how biomedical research and healthcare communities should understand its intension and goals.

The two fields, genomics and bioinformatics – born together with the Human Genome Project (HGP) – have been facing enormous challenges and chances in the past three decades; even more, they, together with other emerging “omics” fields, will have to readjust their prospective as many PM projects are going along to produce the long-promised BIG DATA. To make sense of the data that are not only big but also unprecedentedly diverse in a timely fashion, bioinformaticians are pushed to the frontier.

In order to be prepared for pulling-acts-together, the 5th Young Bioinformatics Principal Investigator (PI) Workshop 2016 has been recently organized by Beijing Institute of Genomics and Beijing Institutes of Life Science, Chinese Academy of Sciences in Beijing, China. Focused on bioinformatics with its applications to big data integration and mining in cutting-edge studies, the Workshop attracted more than 80 field experts, providing an effective platform for in-depth discussion for the roles and emerging issues of bioinformatics in PM. We summarize opinions from the participants here (Opinions and their authorship are listed according to the receiving order) and hope that further brainstorming and discussion will nurture new initiatives and collaborations among the concerned parties.

## Yu Xue (xueyu@hust.edu.cn)

### KEYWORDS: Protein- and PTM-related database and resource; Computational algorithm and tool; Protein–protein interaction and PTM network; Proteomic and PTMomic data

For a better support of studies in PM, the variation, dynamics, and plasticity of protein expression, as well as post-translational modifications (PTMs) must be considered as important layers of the multi-dimensional omics data, which are systematically characterized in individuals and computationally analyzed using bioinformatics approaches. This is because most of biological processes are directly carried out by proteins but not DNAs or RNAs, whereas PTMs play essential roles in the regulation of protein functions. For instance, phosphorylation, one of most well-studied PTMs, is catalyzed by protein kinases. Protein kinases are potent drug targets, which account for 70%–80% of total targets approved for treatment of complex diseases such as cancer. In addition, non-synonymous genetic variations or mutations in coding regions can alter amino acid compositions so as to influence protein PTM states, rewire PTM networks, and potentially affect disease susceptibility in individuals. In this regard, the development of protein- and PTM-related databases and resources, the design of computational algorithms and tools for accurately predicting PTM substrates and sites, the modeling and analysis of protein–protein interaction (PPI) and PTM networks, and the identification of potential drug targets and biomarkers from proteomic and PTMomic data have been posing great challenges for bioinformaticians. Also, the proteomic and PTMomic data must be combined and analyzed together with other types of omics data, to provide a more integrative biomedical knowledge network in the framework of PM.

## Eric-Wubbo Lameijer (eric_wubbo@hotmail.com), Kai Ye (kaiye@xjtu.edu.cn)

### KEYWORDS: Genomic variant; Comprehensive variation profile; Variant calling; Variant discovery

PM consists of two stages: first diagnose precisely, then treat precisely. To diagnose precisely, we need to gather multi-omics data and build a comprehensive profile of an individual. One of the most fundamental and mature forms of omics data is the genomic variant. Genomics has the advantages, not only because genome sequencing is getting cheaper and cheaper, but also because we have knowledge about a huge number of genetic variations corresponding to phenotypes varying from normal cells to disease phenotypes like cancer, due to the enormous amount of past and current genomics research. These differences between individual’s genomes form the starting points of divergence for downstream effects like transcription and translation. It is however still a challenge to effectively capture the full catalog of variations from next- and third-generation sequencing data. Substantial developments are still required, especially for reliably calling germline variants from individuals with inherited diseases, and for calling somatic variants in subclones of tumors, which may have low allele fractions. As the technologies advance, both sequencing and analysis will allow us to not only reliably call variants with a wider size range than is currently possible, but possibly also discover genetic variant types that are missed by current analysis tools yet may impact an individual’s health, leading to discovery of more potential causal variants.

## Kunlin Zhang (zhangkl@psych.ac.cn), Suhua Chang (changsh@psych.ac.cn)

### KEYWORDS: Disease-associated genetic variant; Disease risk evaluation; Functional variant discovery; Multi-dimensional data; Integrative and system-level analysis; Disease-specific genetic architecture atlas

Bioinformatics analysis for disease-associated genetic variants such as single nucleotide polymorphisms (SNPs), copy number variations (CNVs) could play an important role in PM. By now, genome-wide association study (GWAS) and next-generation sequencing (NGS) technology have identified many genetic variants conferring protection against specific diseases. These genetic variants could contribute to PM from different aspects. On January 30th 2015, just 10 days after the US Government announced the Precision Medicine Initiative (PMI), Francis S. Collins, Director of the National Institutes of Health (NIH) and Harold Varmus, Director of the National Cancer Center (NCI), US, wrote a perspective in the *New England Journal of Medicine* entitled “A New Initiative on Precision Medicine” [2]. One important approach of PM mentioned in this “outlook” is genotyping genetic variants of susceptibility genes, which have two applications. The first one is to evaluate disease risk by building evaluation model like polygenic risk score or other machine learning method-based models to precisely guide the disease prevention [3]. The second application of genetic variants is to assist disease treatment by precisely selecting or designing genetic loci-targeted drugs and treatment plan. To fulfill this, bioinformatics analysis, probably combining with biological experiments, to discover functional variants (the variants affect occurrence, development or treatment response) is necessary since most identified variants are only “tags” of functional variants. For this purpose, multi-dimensional data from different levels were needed for the integrative and system-level analysis, including gene expression data, regulatory data, epigenetic data, and pathway/network data. Many bioinformatics methods have been developed for this purpose and we could expect that there will be more to come. On the other hand, besides evaluating disease risk and discovering functional variants based on known susceptibility loci, in future bioinformatics research for this field should continue helping explore genetic variants associated with diseases to complete the atlas of genetic architectures of specific diseases.

## Xiaoyue Wang (pumcwangxy@163.com)

### KEYWORDS: Individualized medical care; Multidisciplinary team; Algorithm development; Data normalization; Unified framework for data integration

PM requires thorough investigation of each individual’s medical and genetic information for the delivery of individualized medical care. One of the big challenges is to integrate different types of data and extract useful information from them for clinical use. The data often include genomic sequences, lab test results, imaging data, and patient’s health records such as demographic data and family medical history. It often takes a multidisciplinary team to work together on it. Bioinformatics, as its name suggests, an interdiscipline to bridge biology and informatics, is therefore an indispensable part in PM. With a knowledge encompassing the computational methodologies, databases, genes and biology, bioinformaticians will work closely with computer scientists and clinicians to embrace the challenges in the following areas: to develop fast and accurate algorithms to process genomics data in order to catch up with the speed of data production; systematical methods to remove the noises from the omics data and proper normalization of different data types; a unified framework to facilitate integration of heterogeneous data, including ontology-based frameworks for electronic health records.

## Jianmin Wu (wujm@bjmu.edu.cn)

### KEYWORDS: Molecular cancer classification; Personalized treatment; Interrogation of heterogeneous data; Best practice of data analysis; Clinical sequencing; Coordinated multidisciplinary efforts

PM is reshaping the landscape of biomedical and clinical research. For example, it is moving into an era by which the tumors are characterized and treated based on their genomic profiles rather than the tissue of origin. The driving force behind this transition is the accumulation of identified mutations, structural variations, epigenetic aberrations, as well as dysregulation of mRNA expression, protein expression, and PTMs, from numerous omics studies. A few of these studies have already led to novel molecular classifications of cancer, which present new opportunities for the personalized treatment. However, the implementation of PM poses substantial challenges for bioinformatics, due to the heterogeneous nature of – omics data and the need to interrogate multiple layers of – omics data simultaneously. Bioinformaticians are needed to work alongside statisticians to develop specific algorithms, databases, and visualization tools for data analysis and integration. Besides, additional obstacles present in translational research, such as the lack of best practice of data analysis for NGS in clinical diagnostics and the inconsistence of data formats of clinical information, would need to be tackled by the coordinated efforts among bioinformaticians, biologists and clinicians.

## Ge Gao (gaog@mail.cbi.pku.edu.cn)

### KEYWORDS: China Precision Medicine Initiative; Nationwide Bioinformatics data infrastructure; Population-tailored reference dataset; Biomedical knowledgebase; Data-rich science

As biology is increasingly turning into a data-rich science, massive data generated by high-throughput technologies pose both opportunities and serious challenges. Powerful bioinformatics infrastructure is critical to store, manage, and analyze these data, and finally to extract novel knowledge effectively and efficiently. This is especially true for PM which aims to providing “customized” healthcare by taking into account individual variability in genes, environment, and lifestyle for each person (https://www.nih.gov/precision-medicine-initiative-cohort-program). One particular challenge in current China is a nationwide data infrastructure which is capable of integrating and curating millions of genetic and molecular data generated by the China Precision Medicine Initiative (CPMI) in the coming five years with various biomedical knowledgebases. Such infrastructure will not only provide a one-stop portal for scientists and clinicians, but also enable a population-tailored reference dataset for Chinese which is critical for data interpretation, clinical/pharmacogenetic marker identification and drug development.

## Fangqing Zhao (zhfq@biols.ac.cn)

### KEYWORDS: Genomic structural variation; Repetitive sequence; Algorithm and tool; Sophisticated model; Heterozygosity rate; *de novo* SV detection; Inherited disease

Genetic variation is the genetic difference both within and among populations, ranging from single nucleotide changes to large-scale karyotypic alterations, which is the genetic basis of phenotypic variation. Recently, extensive studies have shown that genomic structural variation (SV) is involved in various human genetic disorders. As a key technique in PM, SV detection has been proven to be one of the most efficient ways to screen candidate genes related to diseases. Detection and annotation of SVs including indels, duplications, translocations, and inversions, however, are much less straightforward due to the complex structure of certain types of SVs and the repetitive nature of eukaryotic genomes. Many existing multi-signal based tools focus on increasing sensitivities through a combination of all available information, which works well in recognizing true variants with weak but concordant signals [4]. It is necessary to develop new computational algorithms and tools for identifying SVs associated with repetitive sequences and recognizing their precise breakpoints [5]. In addition, more sophisticated models are required to estimate the heterozygosity rate and to filter false positives, which will help detect *de novo* SVs and homozygous deletion variants from personal genomes with inherited diseases. Such development will facilitate the discovery of SVs and susceptibility genes present in our genomes and change our perspective on DNA SVs and human disease.

## Jian Li (Jianli2014@seu.edu.cn)

### KEYWORDS: Driver gene or mutation; Druggable target; Data analysis speed; Collaboration and communication; Gap between data interpretation and clinical practice; Instructive information

PM is established on the basis of our deepened understanding of the human genome and fast accumulation of biomedical data. As a new medical model, it brings us new hope to cure many complex diseases including cancer. However, we are still at primary stage of this revolution and several barriers prevent us from fully benefiting from this great change. (1) We still lack sophisticated tools to accurately identify driver genes or mutations that push disease progression. Even the coding sequences, the best-investigated part of the human genome, remain to be thoroughly understood. Too few mutations identified in coding sequences can serve as drug target. For noncoding sequences, the situation is even worse; most of the noncoding sequences are ‘dark matter’ for us. Thus we need better tools to reveal druggable targets for precise treatment. This goal requires close collaboration between bioinformaticians and molecular biologists. We could then better appreciate the mechanisms underlying complex diseases and confirm targetable molecules through combining findings from clinical sequencing with experimental validation using simplified diseases models (*e.g.*, animal models of human disease). (2) Speed is a necessary factor for clinical practice. For many acute diseases, their progressions are so fast that most of current bioinformatic tools are not able to be competent enough to provide any information for clinical decision in time. We need more powerful hardware and software to speed up the process of data analysis and eventually meet the need of fast clinical decision. (3) Communication between bioinformaticians and clinicians is vitally important. Clinicians face patients and perform clinical practice based on the information provided by bioinformaticians. Bioinformaticians face data and make a judgment on which biomarkers and therapeutic targets can be used for clinicians. Both sides together composite a complete PM system. As bioinformaticians, we need to provide clinicians with clear, understandable and instructive information. The gap between data interpretation and clinical practice should be bridged. This requires bioinformaticians to well understand clinical practice and communicate timely with the clinicians in a mutually understandable language.

## Chunsheng Han (hancs@ioz.ac.cn)

### KEYWORDS: Cause−effect relationship; Functional screening; Goal definition; Biochemical/clinical readouts; Mechanisms of clinical questions; Collaboration

PM requires a deep understanding of the cause–effect relationship between genes and phenotypes. Historically, chemical and physical mutagenesis-based genetic screenings have been the major tool for this challenging task. However, it is painstaking to identify genes responsible for the interested phenotypes even if the clinically-relevant phenotypes can be luckily modeled. Since the accomplishment of HGP and other similar genome projects, as well as the HapMap Project and its related ones, association studies such as GWAS have been widely used as a key step to establish hypotheses about the biological functions of genes. But the following functional investigations are still time-consuming case-by-case adventures. Fortunately, we are now armed with the seemingly ultimate perfect weaponries such as high throughput RNAi- and CRIPSPR-Cas9-based functional screening tools that are both powerful and efficient. The related bioinformatic software for shRNA and gRNA designs and for experimental result evaluations have also been available for researchers although their improvements are still undergoing. The most critical step for large-scale screenings is the clear definition of the biological and clinical goals and the biochemical/clinical readouts that can be monitored or measured by using sophisticated instruments that are easily accessible to most researchers. To this end, good understanding of the underlying cellular and molecular mechanisms of clinical questions is a must. Therefore, bioinformaticians are transforming themselves from pure data analysts into real experts on the biological and medical questions they are interested in and are required to collaborate with clinicians in a more and more intimate manner.

## Shuhua Xu (xushua@picb.ac.cn)

### KEYWORDS: Population genomics; Genetic admixture; Genomic diversity; China Precision Medicine Initiative; Regional effort; National data infrastructure; Genetic structure of population

The genetic background of an individual is a crucial factor to be considered toward personalized medicine or PM. It is now very feasible to re-sequence an individual genome due to recent advances in NGS technologies. However, identifying and prioritizing disease-associated causal variants relies on understanding the distribution of genetic variation within and between populations. In this context, population genomics plays a vital role in dissecting genetic architecture of complex traits/diseases by separating locus-specific effects from genome-wide effects, it is thus a bridge from evolution to medicine. Over the past decades, many joint forces based on international collaborations have made remarkable achievements in the studies of human genetic variation, such as the HGP, HapMap Project, Pan-Asian SNP Project, and the 1000 Genomes Project. Nonetheless, considering very heterogeneous ethnic groups and large population size in China, regional efforts are necessary to provide a more precise and comprehensive characterization of the genomic diversity. In addition, high mobility of people in recent history and modern society considerably increased the chance of inter-ethnic marriages, or genetic admixture, which in turn influences genome diversity and further affects phenotypes relevant to health. Good news is that the Chinese Government has launched CPMI, which is expected to produce vast omics data at both individual and population levels at increasingly faster rates in next 5–10 years. However, not having a well-established national data infrastructure means that we are not really ready to handle issues of data curation, standardization, integration or utilization, *etc*. Furthermore, lack of adequate knowledge of genetic structure of populations increases the risk of failure of study design for those ongoing sampling expeditions supported or not supported by CPMI.

## Jingfa Xiao (xiaojf@big.ac.cn)

### KEYWORDS: Cohort study; Healthy cohort; Disease cohort; Data diversity and integration; PM knowledgebase; Health maintenance; Disease treatment

According to NIH, PM is an innovative approach for disease prevention and treatment, which takes more consideration of individual differences in genes, environments, and lifestyles. Patients share a similar set of symptoms may need different treatments due to their distinct genetic variants. For example, there are many causes of lung cancer, but only people who have a variant within epithelial growth factor receptor (EGFR) respond to treatment with tyrosine kinase inhibitors [6]. Cohort study is a very effective approach for PM. For instance, the well-known Framingham Heart Study that was initiated in 1948 has made substantial contributions to understanding cardiovascular and metabolic diseases [7]. The Nurses’ Health Study (NHS) that was established in 1976 [8] has investigated into the risk factors for major chronic diseases in women, such as cardiovascular disease and breast cancer, whereas another cohort study called the Health Professionals Follow-up Study (HPFS) [9] is all-male study complement to the all-female study NHS. These cohorts help uncover links between lifestyles and cancer, heart disease, as well as other vascular diseases. The diverse data from cohort studies, such as medical records, the patient’s genome, epigenome, proteome, metabolome, environmental, and lifestyle data (https://www.nih.gov/precision-medicine-initiative-cohort-program), may build strong support for future health research. China has been developing several plans for creating and managing large research cohorts, such as healthy cohorts in different areas, major disease cohorts and rare disease cohorts over the next few years. One challenge for bioinformaticians is how to effectively integrate these data and build PM knowledgebase. These knowledge bases may integrate gene, pathway, disease, symptom, pharmacon, diagnosis, and treatment information. Information from knowledgebases will provide a more effective way for researchers working on health maintenance and disease treatment.

## Xuerui Yang (yangxuerui@tsinghua.edu.cn)

### KEYWORDS: Heterogeneity; Individualized therapeutic strategy; Disease-driving pathway; Patient-specific mechanism of disease; Patient classification; In and off label usage of drug; Collaborative effort

PM by its nature requires high-coverage and accurate profiling of a patient’s genetic, epigenetic and other pathological background, which defines classifications of the patients. However, due to the hierarchical structures of the genetic and epigenetic alterations and the heterogeneity across patients, it remains as a challenge to obtain precise and comprehensive patient classifications that can be smoothly translated into individualized therapeutic strategies, especially for the complex diseases such as cancer. One opinion, which I fully agree, is that we need to go beyond the genetic and epigenetic profiling and classify the patients by their mechanisms of disease (MoD). This is based on the following facts. (1) The heterogeneous genetic and epigenetic spectrums usually converge into limited numbers of disease-driving pathways. (2) While the genetic screenings of pre-defined loci over-simplify the classification, the global-level screenings suffer from distractive non-disease-driving alterations, making it highly beneficial to incorporate the MoD for preclusion of these potential false positives. (3) Many of the upstream genetic alterations are currently undruggable, and elucidation of the MoD would not only facilitate classifications of the patients, but also guide the search for applicable in and off label usage of drugs. The next question is how to infer the patient-specific MoD from the noisy multi-omics profiles and use this information for precise and translatable classifications of patients. The solutions rely on collaborative efforts from bioinformaticians, systems biologists, molecular biologists, and last but not the least, clinicians.

## Xiaomin Ying (yingxm@bmi.ac.cn)

### KEYWORDS: Cohort; Biological data; Clinical data; Bioinformatics; Data to medical decision; Knowledge mining; Multi-omics and multi-modality data

**Figure 1:**
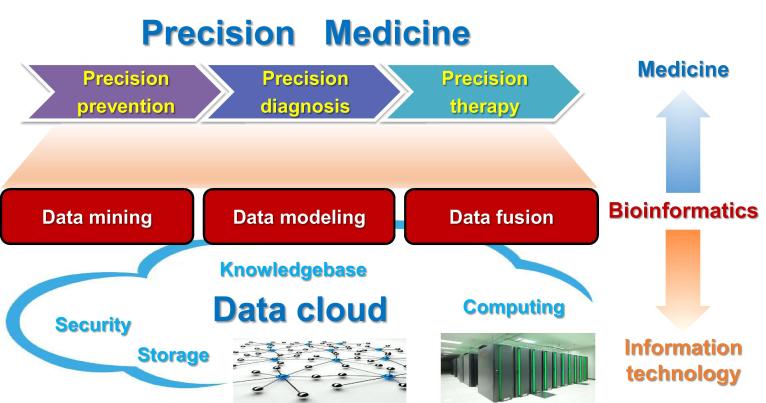
The knowledge framework of Precision Medicine Precision Medicine is built upon big biological and clinical data of large cohorts of patients and healthy controls. Bioinformatics covers all the processes from data analysis to medical decision.

PM comprises three stages, namely precision prevention, precision diagnosis, and precision therapy, which all rely on biological and clinical data of large cohorts of patients and healthy controls. Biological data include genomics, transcriptomics, epigenomics, proteomics, metabonomics, and other omics data. Clinical data include histopathological data, radiography images, and other medical records. The storage, pre-processing and security of these big data rely heavily on information technology (IT). The ultimate medical treatment of prevention, diagnosis, and therapy are mostly handled by clinicians. All the intermediate processes from data to medical decision belong to the scope of bioinformatics. In other words, bioinformatics is a bridge from data to clinic, which makes it one of the most important components in PM. Bioinformatics also faces big challenges in PM. To discover medical knowledge and finally make medical decisions in clinic, bioinformatics has to mine knowledge and build models from big, multi-omics and multi-modality data. Novel mining methods, novel modeling, and data fusion algorithms should be developed to meet the requirements of PM. Opportunities co-exist with challenges. This is the best times for bioinformatics, since PMI will produce unprecedented big data which are abundant ingredients for making delicious food. This is also the worst time for bioinformatics, since it confronts all sorts of data and tries to find the most valuable things to improve people’s health (Figure 1).

## Xuegong Zhang (zhangxg@tsinghua.edu.cn)

### KEYWORDS: Big data; Data integration; Data to knowledge conversion Bioinformatics method; Methodology and theoretical development; Artificial intelligence; Machine learning

The quick accumulation of massive biological and medical data has brought us great hope in people’s campaign against diseases especially for complexes diseases like cancer and rare genetic diseases. Big omics data have shown high potential toward this goal, but to make this goal achievable, there is a huge need for efficient and accurate bioinformatics methods to process and analyze various types of existing and emerging omics data, for efficient and reliable methods to handle and process phenotypic data including medical records and data from various types of equipment, and for powerful methods that can convert data to information, and discover knowledge from information. Technologies for obtaining biological and medical data such as DNA sequencing, medical imaging, and phenotype description at clinics are far from perfect. Both the volume and complexity of biological and medical big data have far exceeded the capability of human beings. Due to this nature of the data, just the collection of big data or even the systematic integration of big data does not automatically lead to breakthroughs in PM. Methodology and theoretical development becomes even more crucial than ever to fully realize the potential in the big data. Recent successes of artificial intelligence (AI) especially machine learning in the IT industry and in playing games with human have demonstrated the great power of applying advanced machine learning methods on big data for solving complicated problems. Machine learning has been playing important roles in methods for functional genomics and systems biology, and will play more crucial roles in the integrative analysis of mixed biological and medical big data. It can be expected that advanced research on machine learning and other AI methods and technologies for analyzing big bioinformatics and medical informatics data will be a key component in all efforts toward PM.

## Wei-Hua Chen (weihuachen@hust.edu.cn)

### KEYWORDS: Clinical outcome; Disease risk prediction; Diverse genetic and environmental backgrounds; Capture of disease-contributing factors; Multidisciplinary collaboration

One of the critical features of PM is its ability to tailor the preventive measures and medical treatments to the phenotypic and genotypic characteristics of each patient in order to obtain the best clinical outcome. Obviously, the best clinical outcome one can get is not to get any diseases in the first place. To achieve this, researchers first of all need to build predictive models by using all available information pertaining to specific subjects including family history, personal disease history, genomic mutational profile, gene expression profile, a variety of related biometric measurements, and exposures to environmental risk factors. The information will have to be collected from a large number of individuals with diverse genetic and environmental backgrounds so that all possible variations in factors contributing to the development of the diseases can be captured. Then in order to accurately predict disease risks, individuals’ genetic and biometric information will have to be regularly monitored, analyzed, and evaluated. PM thus calls for collaboration from experts in the fields of bioinformatics, big data, machine learning, data minding, cloud computing, mobile health, and many others. Opportunities exist in every step of the way for the academia, medical institutions, and industry, so do challenges.

## Yun Liu (yliu39@fudan.edu.cn)

### KEYWORDS: Practice of medicine; Big data; Data sharing; Institutional barriers; Data system compatibility; Data standards; Patient privacy; Data regulation

PM, which promises the “delivering the right treatment at the right time, every time, to the right person.” as said by President Obama when he announced PMI, is transforming the healthcare system and will, ultimately, change the practice of medicine. In order to fulfill this promise, we rely on big data, including clinical records, multi-omics data, lab-test results, and imaging data, from which disease-specific patterns can be identified and used to provide specific treatments unique to each individual. Thus, data sharing is critical for achieving the goal of PM. However, there are three major challenges for data sharing: (1) institutional barriers. Data have been viewed as a commodity these days, and groups with larger datasets enjoy a competing advantage over groups with smaller ones. However, these barriers will eventually hinder mutual aids and communal breakthroughs. (2) Incompatible data systems. We need to make sure that our systems can communicate. Today, patient data are usually inaccessible and rarely documented in any usable way. Moreover, different institutions may adopt different data standards, which makes it almost impossible to integrate information across different systems. (3) Patient privacy. Fears about privacy, confidentiality and cyber-security must be addressed, so that regulatory issues won’t hamper innovation. Even though none of these challenges are simple to surmount, the scientific community has realized that data sharing is essential for achieving the goal of PM. The practice of sharing big data from many large collaborations among multiple institutions aboard has provided invaluable experience for us and will eventually facilitate progress.

## Zhang Zhang (zhangzhang@big.ac.cn)

### KEYWORDS: Big data curation; Funding support; Computational methods; Worldwide interdisciplinary communications & collaborations; High-performance computing technologies; National data infrastructure

Advancements in high-throughput sequencing technologies as well as high-performance computing technologies accelerate biomedical research entering into a new era of PM, with the ultimate goal to develop more effective ways for precision healthcare and personalized medical treatment [2]. To achieve this goal, we need to address several critical challenges that result primarily from big data, including not only omics data but also a wide range of health data (such as clinical data, physiological data, and environmental data). One of the most challenging issues is big data curation, that is, to enhance data interoperability and consistency by the process of standardization, quality control, and annotation. Although computer programs can aid, to a certain degree, to realize automated curation, curation is heavily dependent on human resources and is largely done by dedicated experts as well as the scientific community (that is, expert curation and community curation, respectively) [10]. Therefore, data curation as well as its sustainable funding support can increase the promise to obtain high-quality data that is critical to perform downstream data analysis, with higher precision. Without high-quality data, nothing could be achieved or at least it is not precise enough for personalized healthcare or treatment. Second, computational methods that aim to associate omics data with health data, decipher the underlying mechanisms of genetic variations, develop precise personalized healthcare and prevention strategies, and dig out potential biomarkers for disease treatment are badly needed. To make it achievable, worldwide interdisciplinary communications and collaborations among biologists, physicians, curators, bioinformaticians, IT developers, and others are very crucial. In addition, high-performance computing technologies (*e.g.*, cloud computing, Hadoop/Spark) are desirable to be employed to speedup bioinformatics tools that are often used to deal with large-scale datasets [11]. Third, a national-level infrastructure with powerful computing resources is a must. PM implies big data and big data supports precision research. Therefore, all spectrums of data operations (including deposition, integration, curation, and analysis) are becoming increasingly daunting and more time-consuming, and thereby are impossible to be achieved in a single laboratory or institution, particularly considering the fact that biomedical data keep growing at much higher rates.

## Kun Huang (kun.huang@osumc.edu)

### KEYWORDS: Data security; Privacy protection; Data ownership; Ethical and legal issues; Genetic counseling support; Benefit for contributing patients

The development of PM calls for integration of multiple types of omics, imaging, and clinical data from large cohorts of patients for different diseases. While advancement in informatics methods tackling the algorithmic and infrastructure aspects of PM will undoubtedly result in discovery of new biomarkers and therapeutical schemes, issues on data security, privacy protection, and data ownership will lead to new challenges and hurdles on the success of such studies. Currently, many of these problems are still being debated and solutions for them are being explored. Nevertheless, it is critical that these issues are taken into full consideration during both study design and implementation stages as well as patient recruiting and consenting processes to ensure sustainable operation of the research programs. In addition, given the complicated ethical and legal issues involved in generating, sharing, and consuming genetic data, the patients should be provided with strong genetic counseling support. Last but not the least, while many of the large cohort studies will benefit future patients, an interesting problem is how to benefit patients who contributed data to these studies. An example is the Total Cancer Care Program adopted by multiple cancer centers in US. This protocol allows consented patients to be contacted for clinical trials based on their genomic data.

## Jun Yu (junyu@big.ac.cn)

### KEYWORDS: Patient-centric; Cohort study; Longitudinal data collection; Knowledge sharing and integration; High-resolution tools; Convergence of frontier technologies; Collaboration

As the real sequel of HGP in both priority and scale, the PM projects, including versions proposed among various nations, will certainly have to pass on the legacy of HGP. (1) HGP has a defined goal, which is to assemble a single complete human genome sequence; PM projects also have their defined goals: to build specialized databases from millions of patients and cohorts. (2) PM projects drive to build new research infrastructures, including emerging research fields and joint research programs between basic and clinical research communities. (3) PM projects should recognize key technologies and continue to fund them, such as single cell manipulation and direct RNA sequencing. (4) PM projects should promote technology development and novel applications in relevant industries, such as assays for large-scale drug screening and novel drug targets.

PM will not only change our research focuses and priorities but also our ways to do research. (1) PM projects are definitely patient-centric, where diseases and population-based cohorts are where to start. (2) PM research activities are moving toward common diseases in addition to genetic or rare diseases. (3) All research projects need to be extended toward the temporal direction in terms of sampling and following-up studies. (4) All tools have to be sharpened toward their ultimate resolutions; single-molecule and single-cell technologies are certainly the rules not exceptions. (5) One obvious challenge for the life science/biomedical research communities is to unite the professional language and conception as well as to promote efforts and collaborations among four divergent research domains: the anatomy–physiology, the cellular–molecular biology, the genotype–phenotype, and omics–system domains. In addition, a set of common elements have to be extracted for knowledge sharing and integration. Finally, in order to do a best job for PM, we have to recognize the necessity of converging frontier technologies from other scientific disciplines and fields, especially microelectronics, microfluidics, nanotechnology, and artificial intelligence. If we have to point out one of them for which we have to muster, it is to sequence RNA directly at single molecule level.

## Competing interests

The authors have declared that there are no competing interests.
